# Androgen receptor acts as the transcriptional repressor of the nuclear receptor LRH-1 via the androgen-driven chromatin looping conformation in prostate cancer

**DOI:** 10.1016/j.gendis.2025.101903

**Published:** 2025-10-24

**Authors:** Wenxing You, Tiantian Gao, Haolong Li, Daniel Hau Tak Lam, Wenjuan Xie, Lijia Xiao, Weijie Gao, Dinglan Wu, Junjian Wang, Yuliang Wang, Franky Leung Chan

**Affiliations:** a School of Biomedical Sciences, Faculty of Medicine, The Chinese University of Hong Kong, Shatin, Hong Kong 999077, China; b Department of Surgery, Faculty of Medicine, The Chinese University of Hong Kong, Shatin, Hong Kong 999077, China; c Department of Anatomical and Cellular Pathology, Faculty of Medicine, The Chinese University of Hong Kong, Shatin, Hong Kong 999077, China; d Department of Clinical Laboratory Medicine Center, Shenzhen Hospital, Southern Medical University, Shenzhen, Guangdong 518052, China; e School of Pharmaceutical Sciences, Sun Yat-sen University, Guangzhou, Guangdong 510006, China

**Keywords:** Androgen receptor, Castration-resistance, Chromatin looping, LRH-1, Prostate cancer

## Abstract

The androgen receptor (AR) signaling axis is regarded as the key driver of prostate cancer (PCa). Besides acting as a well-characterized transactivator of diverse targets, accumulating evidence suggests that AR can also function as a transrepressor. However, AR-repressed targets and their significance in PCa and castration-resistant PCa (CRPC) remain poorly understood. Among multiple mechanisms, intratumoral androgen biosynthesis is regarded as an important factor responsible for persistent AR signaling in CRPC. Previously, we characterized that the nuclear receptor LRH-1 (*NR5A2*) plays a key role in the promotion of intratumoral androgen biosynthesis in CRPC via its direct transcriptional control of multiple key steroidogenic enzymes. However, the transcriptional control of LRH-1 in PCa is still undefined. In this study, we show that androgen-activated AR could suppress, whereas antiandrogen-suppressed AR could up-regulate the LRH-1 expression in PCa cells. Furthermore, our genomics analysis showed that the transcriptional repression of *NR5A2* by ligand-activated AR was mediated through the induction of a distinct androgen-dependent chromatin looping formed within the topologically associated domain of *NR5A2* via direct binding of AR to the regulatory elements of *NR5A2*. Our present study demonstrates the significance of decreased androgen levels in androgen-deprivation therapy, resulting in the relief or up-regulation of LRH-1 toward intratumoral androgen biosynthesis in CRPC.

## Introduction

Androgen receptor (AR) signaling plays a crucial role in the development and progression of prostate cancer (PCa).^1^ Hormone or androgen-deprivation therapy (ADT), which primarily acts to suppress the AR activity via either androgen depletion or AR antagonists, is still the preferred standard treatment option for locally advanced and metastatic prostate cancer.^2^ Although it is initially effective, most patients still inevitably develop resistance to therapy and have tumors that relapse to castration-resistant PCa (CRPC) within a few years. Among multiple AR-dependent or -related mechanisms, intratumoral androgen biosynthesis is regarded as an important factor responsible for the CRPC growth, as it can significantly contribute to sustaining the relatively high tumor androgen levels and the persistent AR signaling, despite low castrate serum levels of androgen upon the systemic ADT or castration.^3^^,^^4^ One important mechanism mediating the synthesis of intratumoral androgen is the up-regulation of many steroidogenic enzymes expressed within prostate tumor cells that convert weak adrenal androgens into the high-affinity AR ligands testosterone (T) and dihydrotestosterone (DHT).^5^ However, how this enzymatic machinery is dysregulated in CRPC remains to be characterized.

Liver receptor homolog-1 (LRH-1, encoded by *NR5A2*) belongs to the NR5A subfamily of the nuclear receptor superfamily. It was originally identified as a transcription factor regulating a significant number of genes functionally involved in liver metabolism and in the development and metabolism of multiple endoderm-derived organs.^6^ Recent studies have also suggested that LRH-1 plays a role in the pluripotency of stem cells,^7^ and performs various oncogenic functions in a range of cancers, including prostate and breast cancers.^8^, ^9^, ^10^ Previously, we showed that LRH-1 can play a significant role in the growth of CRPC via its direct transcriptional control of multiple key steroidogenic enzyme genes in promotion of intratumoral androgen biosynthesis.^8^^,^^11^ However, its transcriptional control in PCa is still largely undefined.

Much of our current understanding on AR signaling is largely based on the established transactivation function of AR, particularly its positive regulation of genes involved in the development and growth of the prostate gland and PCa, via its binding to specific androgen–response elements (AREs) in the regulatory regions of AR-target genes followed by the recruitment of coactivators and transcription initiation.^12^^,^^13^ On the other hand, accumulating studies have shown that ligand-activated AR can also function as a transcriptional repressor, including itself, action of which involves both genomic (via its direct binding to DNA) and epigenetic (interactions with histone modifiers) mechanisms.^12^^,^^14^, ^15^, ^16^ A few ChIP-based whole-genome sequencing studies have identified a significant number of AR-repressed target genes in PCa cells, further suggesting that AR can also function as a transcriptional repressor.^17^^,^^18^ These AR-repressed genes, as identified individually in PCa cells, can perform multiple tumor-suppressing or oncogenic functions, involved broadly in cell adhesion, cell cycle progression, cell growth and proliferation, and inactivation of androgens.^14^^,^^15^ However, the significance and transcriptional regulation of these AR-repressed genes in PCa progression remain poorly understood. The restoration of some of these AR-repressed genes (particularly *SOX2, BRN2* and *PEG10*) during intense ADT is suggested to play roles in CRPC and neuroendocrine prostate cancer/NEPC progression.^19^, ^20^, ^21^, ^22^

It is established that the spatial organization of the genome or 3-dimensional (3D) chromatin structure has a significant impact on the proper cell-specific transcription regulation and biological functions, which depends on the spatial interaction between the distal enhancers and proximal promoters via long-distance chromatin-looping.^23^ The 3D chromatin structure is usually organized as the topologically associated domains (TADs), the structural and functional units of chromosomes, which can be transcriptionally active or repressed.^24^^,^^25^ Disruption of enhancer–promoter loops within particular TADs can lead to gene dysregulation and diseases, including cancers.^26^ AR can bind to some specific *cis*-regulatory elements (CREs), located distally to gene promoters. These AR-bound CREs can be brought to close proximity with promoters via chromatin loop formation and function as enhancers in long-range gene regulation via epigenetic modifications.^27^^,^^28^ However, the impact of AR-CREs on the transcriptional activation and repression of their target genes via chromatin-loop formation in PCa is still unclear.

In this study, we investigated AR signaling in the regulation of LRH-1 expression in PCa. Here, via genomics analysis, we showed that LRH-1 (*NR5A2*) is a direct AR-repressed target in PCa cells via a mechanism of induction of a distinct chromatin looping pattern formed within the topologically associated domain (TAD) of *NR5A2* mediated by the direct binding of AR to the regulatory elements of *NR5A2*. Our results also suggest a mechanism of up-regulation of LRH-1 in CRPC, at least partially due to the release or attenuation of the repression by AR.

## Materials and methods

### Genomics data analyses and annotations

PCa-related genomics datasets of ChIP-sequencing (ChIP-seq), global run-on and sequencing (GRO-seq) and mRNA microarray were obtained from the Gene Expression Omnibus (GEO) for genomics analyses and included the following datasets: GSE14092,^29^ GSE31410,^17^ GSE32269,^16^ GSE28950,^30^ GSE35988,^31^ GSE55062,^32^ GSE59986,^20^ GSE84432^33^ and GSE94682.^34^ For molecular signature analysis via the Gene Set Enrichment Analysis (GSEA), acquired microarray chips or RNA-seq derived gene sets and annotation profiles, sample description files and transcriptome datasets were organized in the format required by GSEA and uploaded to GSEA software for annotation and enrichment analysis.^35^ The ChIP-seq datasets and GRO-seq datasets in BedGraph format were uploaded to the UCSC Genome Browser (http://genome/ucsc.edu/) for sequencing alignments, annotations and nascent RNA synthesis analyses.

### Cell lines and cell culture

Three human PCa cell lines (LNCaP, VCaP and DU145; ATCC, Manassas, VA) were used in this study. The cells were cultured under conditions as described previously.^36^^,^^37^ The LNCaP-AI cell line was generated via long-term culture of the parental LNCaP cells under androgen-deprived conditions and cultured in RPMI-1640 medium containing 10% calf serum-fetal bovine serum (CS-FBS) as described previously.^38^ Information on the culture media used for the growth of these cell lines is provided in Supplementary Information (Table S1).

### Castration-resistant PCa xenograft tumors

The patient-derived xenograft (PDX) TM00298 tumors, purchased from the Jackson Laboratory, were propagated subcutaneously in the dorsal flank of 6- to 8-week-old intact male NSG mice. Host mice bearing xenograft tumors were then castrated by bilateral orchiectomy once the tumor size reached about 0.8 cm^3^. Biopsies of tumors were acquired on the same days when castration was performed (pre-castration), and when tumors grew rapidly to sizes of about 1.2 cm^3^ (castration-relapse) for subsequent protein analyses. The animal experiments were performed following the institute guidelines and with prior approval by the University Animal Experimentation Ethics Committee, CUHK.

### Plasmid construction

(a) Expression plasmid: The AR expression plasmid pLenti6/V5-AR was constructed as described previously.^39^ (b) The shRNA plasmid: pLKO.1-shAR was constructed by inserting the annealed oligonucleotide primers targeting AR into the *Age*I-*Ec*oRI digested pLKO.1-puro vector. (c) Reporter plasmids: fragments of regulatory regions of the human *NR5A2* (LRH-1) gene with AR-binding sites (ARBS) were PCR-amplified with HiFi DNA polymerase using genomic DNA extracted from VCaP cells as a template and inserted into the pGL4 basic vector as various pGL4-ARBS plasmids. The mutants pGL4-ΔARBS3 and pGL4-ΔARBS-promoter with deletions of ARBSs were constructed via the overlap extension PCR method.^40^ The nucleotide sequences of the primers used for plasmid construction are listed in the Supplementary Information (Tables S2 and S3).

### *In vitro* cell growth analysis

The cells were seeded at a density of 2 × 10^3^ cells/well in 96-well plates and cultured in normal media supplemented with CS-FBS at various dihydrotestosterone (DHT) doses. The number of viable cells was determined by a cell counting kit-8 (CCK-8) assay following the manufacturer's procedures (Dojindo Molecular Technologies, Inc.).

### PCR and immunoblot analyses

RT-qPCR and immunoblotting were performed as described previously,^39^^,^^41^ and information on the primer sequences is provided in the Supplementary Information (Table S4). The primary antibodies used include the following: AR (N-20), PSA (C-19) and β-actin (C4) from Santa Cruz Biotechnology and LRH-1 (ab41901) from Abcam.

### Molecular biology analyses

(a) RNA interference. Procedures for the generation of VCaP-shAR cells with stable AR-knockdown were followed as described previously and are described in the Supplementary Information.^42^ (b) Chromatin immunoprecipitation (ChIP) assay. ChIP assays of the endogenous *AR* gene promoter were performed in VCaP cells treated with or without DHT following procedures described previously.^43^ The purified DNA was analyzed via qPCR using primer pairs listed in Supplementary Table S5. (c) Luciferase reporter assay. LNCaP or VCaP cells were pre-cultured with CS-FBS for 24 h before transfection, followed by treatment with DHT or without DHT at 24 h post-transfection. After 48-h DHT treatment, the cells were assayed for luciferase reporter activity via the dual-luciferase reporter method (Promega) as described previously.^43^ (d) Chromosome conformation capture assay (3C). *Dpn*II was selected for enzyme digestion of chromatin after analysis of the digestion map of the DNA region (−336 kb to +3 kb) covering the enhancer-promoter region and intron 1 of the *NR5A2* gene. The 3C assay was performed in VCaP cells following procedures described previously.^44^ Briefly, cross-linked chromatin extracted from VCaP cells was digested with *Dpn*II (400 U) overnight at 37 °C. Eluted chromatin was re-suspended in ligation buffer and ligated with T4 ligase. The re-ligated chromatin was reverse-crosslinked with 100 μg/mL proteinase K and digested with RNase A (10 mg/mL, NEB). The purified DNA was analyzed via standard PCR using primers listed in Table S6.

### Statistical analysis

All data are presented as the mean ± SD from at least three independent experiments. Statistical analyses of normally distributed data were performed using two-tailed Student's *t*-test for replicates (*n* = 3) and one-way ANOVA test for replicates > 3. Differences were considered significant when *P* < 0.05.

## Results

### LRH-1 shows an up-regulation expression in CRPC xenograft and cell models

**Figure 1:**
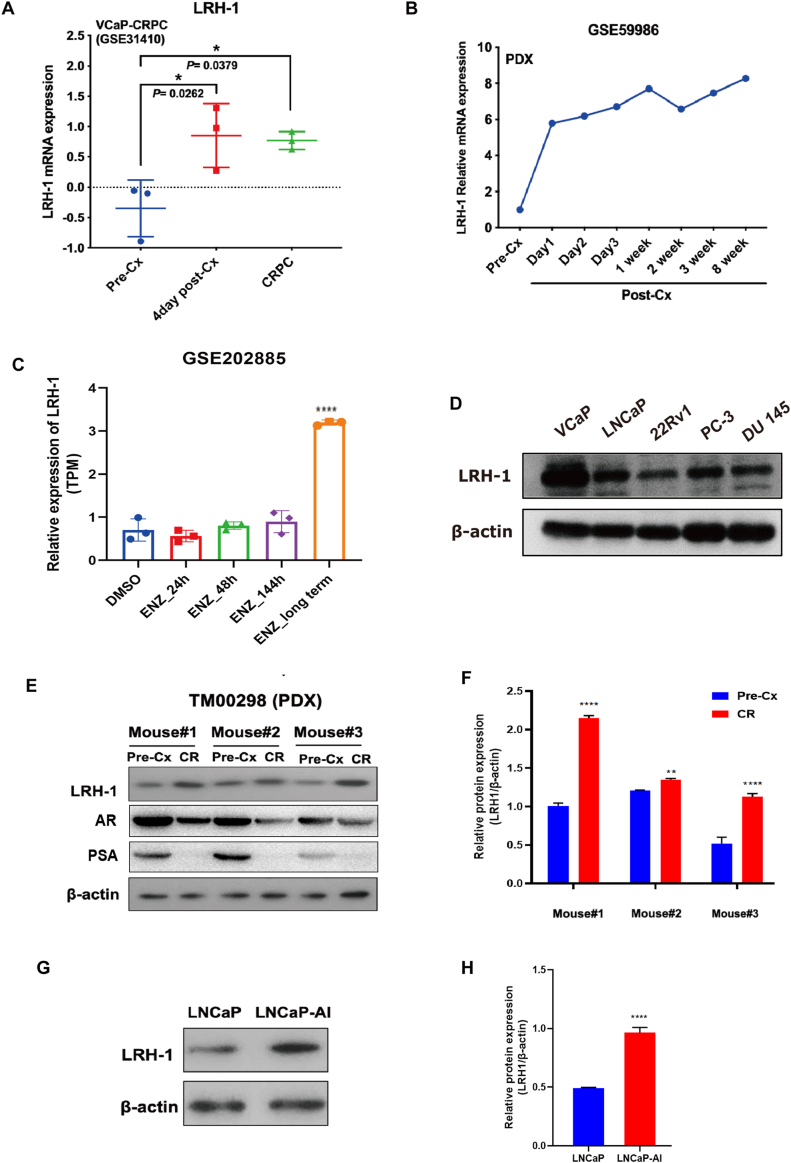
LRH-1 shows an up-regulated expression in CRPC xenograft and cell models. **(A)** Expression profile of LRH-1 in the VCaP-CRPC xenograft model as revealed from a GEO dataset (GSE31410). **(B)** Expression profile of LRH-1 in a patient-derived xenograft (PDX) model of PCa as revealed from a GEO dataset (GSE59986). **(C)** Expression profile of LRH-1 in CRPC PCa cells after long-term anti-androgen treatment (enzalutamide treatment). **(D)** Endogenous expression of LRH-1 in various PCa cell lines. **(E, F)** Immunoblots and the relative protein expression levels of LRH-1, AR and PSA in TM00298-CRPC PDX model. **(G, H)** Immunoblot analysis revealed that LRH-1 displays significantly higher protein levels in the LNCaP-derived androgen-independent cell subline LNCaP-AI than in the parental LNCaP cells. ∗*P* < 0.05; ∗∗*P* < 0.01; ∗∗∗∗*P* < 0.0001.

Previously, we demonstrated that the nuclear receptor LRH-1 displays an increased immunohistochemical signal in high-grade hormone-naïve and CRPC tissues, and its increased expression pattern is validated in two GEO expression microarray datasets of hormone-naïve and CRPC samples.^8^ To further validate the expression profile of LRH-1 during the progression of CRPC, we analyzed two expression microarray datasets of CRPC xenograft models. Analysis of the dataset from the castration-relapse VCaP-CRPC xenograft model (GSE31410) showed that LRH-1 exhibited a significant increased expression in xenograft tumors at day 4 post-castration and maintained at high levels in the castration-refractory period compared with that in pre-castration (Fig. 1A). Similarly, dramatic up-regulation of LRH-1 was also detected in xenograft tumors on day 1 and was maintained at high levels for up to 8 weeks beyond post-castration in LTL331, a patient-derived xenograft (PDX) model of PCa (GSE59986) (Fig. 1B). LRH-1 expression was also elevated after long-term antiandrogen treatment (enzalutamide treatment) in CRPC PCa cells (GSE202885) (Fig. 1C). The endogenous protein expression of LRH-1 in various PCa cell lines is shown in Figure 1D. Moreover, we established a CRPC xenograft model based on the castration-relapse growth of the AR-positive PDX TM00298. Expression analyses of xenograft tumors revealed that the protein levels of LRH-1 displayed significant increased expression; in contrast, the protein levels of AR and PSA exhibited decreased expressions in castration-relapsed PDX tumors grown in castrated host mice compared to those in pre-castration tumors grown in intact mice (Fig. 1E and F). Besides, we examined the LRH-1 expression in the androgen-independent prostate cancer cell line LNCaP-AI (derived from more than 6-month culture of LNCaP cells in charcoal-stripped serum),^38^ and found that LNCaP-AI cells exhibited significantly higher endogenous expression levels of LRH-1 compared to their parental LNCaP cells (Fig. 1G and H). Together, these results indicated that significant up-regulation of LRH-1 was manifested in CRPC and that its expression was promptly elevated in response to castration, as revealed in the CRPC models.

### LRH-1 expression displays an inverse correlation with AR signaling in PCa cells

**Figure 2:**
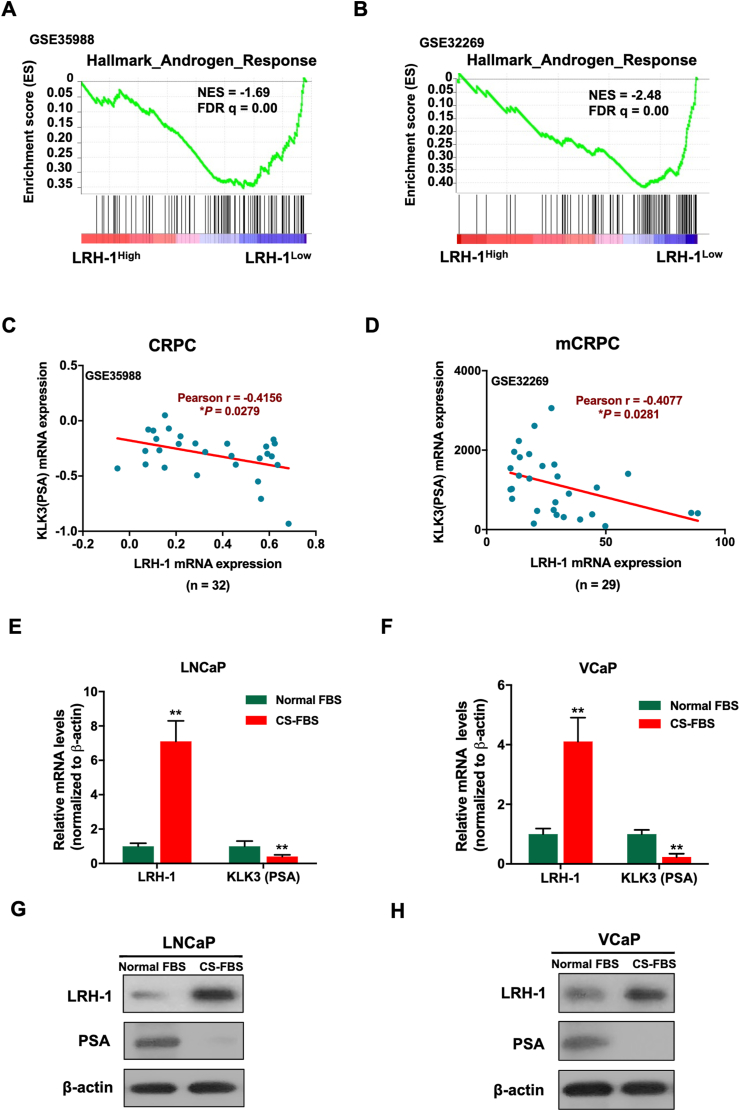
LRH-1 expression shows a negative correlation with AR signaling in prostate cancer. (**A, B**) Gene set enrichment analysis (GSEA) of hallmark genes of the androgen response (ARGs) was performed on the LRH-1^High^ and LRH-1^Low^ subsets of prostate tumors from two GEO datasets (GSE35988 and GSE32269). Prostate tumors were ranked according to their expression levels of LRH-1, with 50% higher values grouped as LRH-1^High^ and *vice versa*. NES = normalized enrichment score; FDR = false discovery rate. **(C, D)** Pearson correlation and linear regression analysis between KLK3 and LRH-1 in CRPC and mCRPC samples using two GEO datasets (GSE35988 and GSE32269). Pearson correlation coefficient, *r* = −0.4; ∗*P* < 0.05. (**E, F**) RT-qPCR analysis of LRH-1 and KLK3 (PSA) expressions in LNCaP and VCaP cells cultured with androgen-deprived CS-FBS medium. (**G, H**) Immunoblot analysis of LRH-1 and PSA in LNCaP and VCaP cells cultured with CS-FBS medium for 3 days. ∗∗*P* < 0.01.

Previously, we showed that LRH-1 can regulate the intratumoral androgen biosynthesis and thus AR signaling in PCa.^8^ We herein investigated whether AR signaling could reciprocally affect the expression of LRH-1. We first analyzed the transcriptome data of hormone-naïve prostate cancer and CRPC derived from two GEO datasets (GSE35988 and GSE32269) and divided the prostate tumors into two subsets, LRH-1^Low^ and LRH-1^High^, according to their relative LRH-1 mRNA levels. GSEA analysis for the hallmark genes of the androgen response was performed between the LRH-1^Low^ and LRH-1^High^ subsets of prostate tumors. The results showed that the androgen-responsive genes (ARGs) displayed a significant negative enrichment in the LRH-1^High^ subset of prostate tumors (Fig. 2A and B), indicating that the expression level of LRH-1 in prostate tumors was inversely correlated with the AR signaling activity. Moreover, the mRNA expression levels of LRH-1 and KLK3 exhibited a negative correlation in CRPC samples by analyzing the same GEO datasets from two clinical cohorts of CRPC and mCRPC (Fig. 2C and D). We further examined the effects of culture with charcoal-stripped serum (CS-FBS; mimicking androgen deprivation) on two AR-positive LNCaP and VCaP cells. The results showed that CS-FBS culture increased the mRNA and protein levels of LRH-1 but decreased the level of KLK3 (PSA) in both LNCaP and VCaP cells (Fig. 2E–H). Together, these results suggest a negative role of androgen or AR signaling in the regulation of LRH-1 expression in CRPC.

### LRH-1 expression is suppressed by androgen but elevated by antiandrogen in AR-positive PCa cells

**Figure 3:**
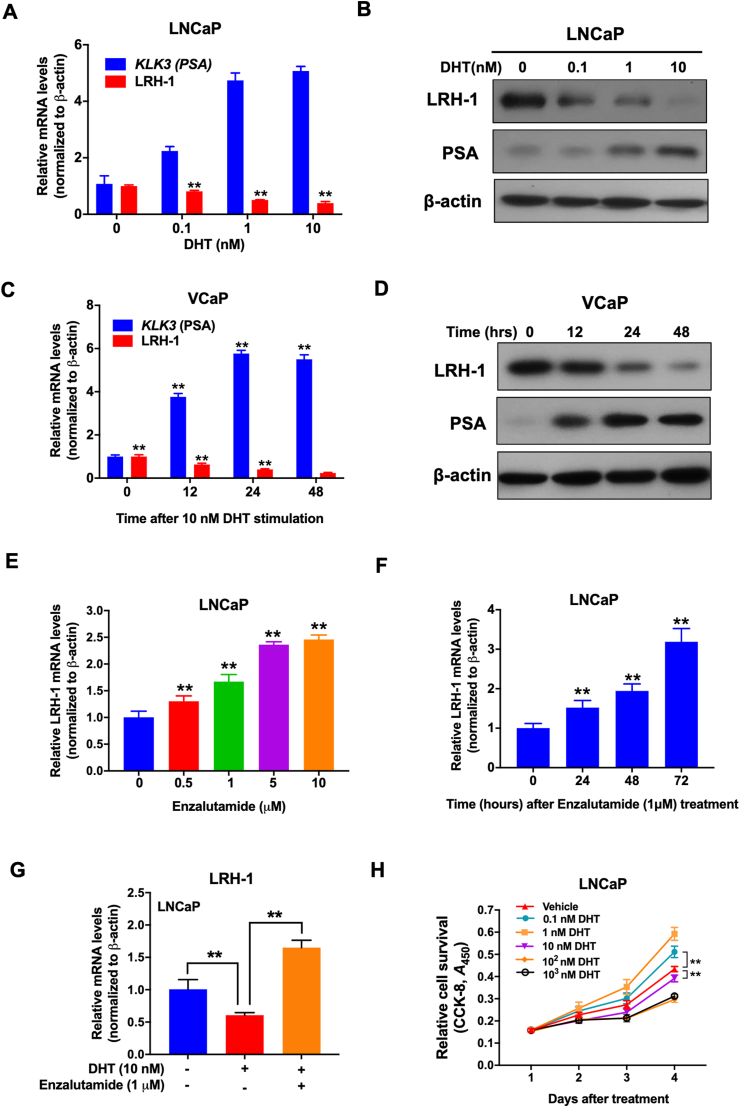
LRH-1 expression is suppressed by androgen but elevated by anti-androgen in AR-positive prostate cancer cells. (**A, B**) RT-qPCR and immunoblot analyses of LRH-1 and KLK3 (PSA) expressions in LNCaP cells upon treatment with a gradient dose of DHT. **(C, D)** RT-qPCR and immunoblot analyses of LRH-1 and KLK3 (PSA) expressions in VCaP cells upon DHT treatment for 0–48 h. VCaP cells were pre-cultured with CS-FBS medium before treatment with 10 nM DHT for duration 0–48 h. **(E, F)** RT-qPCR analysis of LRH-1 expression in LNCaP cells upon treatment with gradient doses of enzalutamide (0–10 μM), and in VCaP cells after treatment for 24–72 h. **(G)** RT-qPCR analysis of LRH-1 expression in LNCaP cells upon combined treatment with DHT and enzalutamide. LNCaP cells were pre-cultured with CS-FBS medium for 72 h before treatment with either DHT alone or DHT combined with enzalutamide for 24 h. **(H)** CCK-8 assay. AR-positive LNCaP cells were treated with 0.1–10^3^ nM DHT. The results showed that low-dose DHT (0.1–1 nM) could promote, whereas high-dose (10–10^3^ nM) could inhibit the cell proliferation of LNCaP. ∗∗*P* < 0.01.

To further determine the effect of androgen on the expression of LRH-1 in PCa cells, we next sought to determine LRH-1 expression in two AR-positive (VCaP and LNCaP) and an AR-negative (DU 145) cells upon DHT treatment. The results showed that DHT treatment for 24 h significantly suppressed both the mRNA and protein levels of LRH-1 but increased the PSA level in LNCaP cells in a dose-dependent manner (Fig. 3A and B). Further analysis on the effect of DHT treatment duration revealed that the suppressive effect of DHT on LRH-1 expression in VCaP cells was prolonged with increasing treatment duration (Fig. 3C and D). However, such a suppressive effect on LRH-1 expression was not detected in the AR-negative DU 145 cells (Fig. S1). Conversely, the suppression of AR activity by the antiandrogen enzalutamide significantly elevated the LRH-1 mRNA expression in LNCaP cells in both a dose- and time-dependent manner (Fig. 3E and F). On the other hand, the suppressive effect of DHT on LRH-1 expression was completely abolished by combined treatment with enzalutamide in both LNCaP and VCaP cells (Fig. 3G; Fig. S2A). Intriguingly, *in vitro* growth characterization analyses showed that low-dose DHT (0.1–1 nM) could promote proliferation, whereas high-dose (10–10^3^ nM) could inhibit the cell proliferation of androgen-sensitive LNCaP but not the insensitive DU 145 cells (Fig. 3H; Fig. S2B). Together, these results suggest that androgen can suppress LRH-1 expression in androgen-sensitive but not androgen-insensitive PCa cells. Androgen at various concentrations can trigger different growth responses in AR-positive PCa cells.

### Androgen-induced suppression of LRH-1 expression in androgen-sensitive PCa cells is mediated through AR

**Figure 4:**
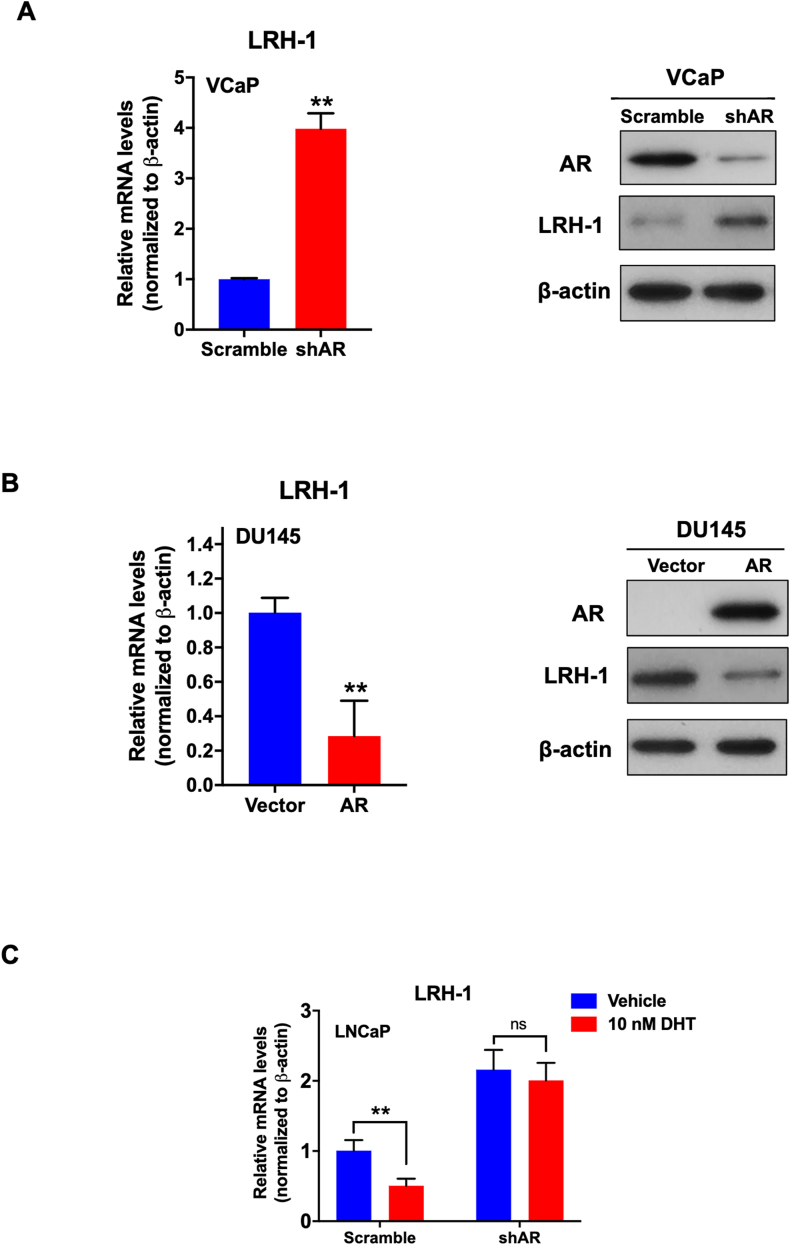
Androgen-induced suppression of LRH-1 expression in androgen-sensitive PCa cells is mediated through AR. **(A)** RT-qPCR and immunoblot analyses of LRH-1 expression in VCaP-shAR transductants with stable AR knockdown. The results showed that a significant increase in LRH-1 mRNA (left panel) and protein (right panel) expression was induced in VCaP-shAR transductants compared with VCaP-scramble transductants. **(B)** RT-qPCR and immunoblot analysis of LRH-1 expression in DU145-AR transductants with stable ectopic AR expression. The results showed that a significant decrease in LRH-1 mRNA (left panel) and protein (right panel) expression was induced in DU145-AR transductants compared with DU145-vector transductants. **(C)** RT-qPCR analysis of LRH-1 expression in LNCaP-shAR transductants upon (10 nM) DHT treatment. The results showed that the DHT-induced suppression on LRH-1 expression was not observed in LNCaP-shAR transductants compared with control LNCaP-scramble transductants. ns: not significant; ∗∗*P* < 0.01; ns: not significant.

To demonstrate whether the androgen-induced suppression of LRH-1 expression in PCa cells is mediated through its cognate receptor, we next generated the stable AR-knockdown transductants in VCaP cells (with high endogenous expression of wild-type AR) and stable AR transductants in AR-negative DU 145 cells for the evaluation of the effects of AR on DHT-mediated suppression on LRH-1 expression in PCa cells. The results showed that stable knockdown of AR induces a significant increase of both the mRNA and protein levels of LRH-1 in VCaP-shAR-transduced cells (Fig. 4A). Conversely, stable ectopic expression of AR induces significant decreases of LRH-1 mRNA and protein levels in DU145-AR transductants (Fig. 4B). Furthermore, the DHT-induced suppression on LRH-1 expression was abolished in LNCaP-shAR transductants with stable AR knockdown (Fig. 4C). Together, these results indicate that the androgen-induced suppression on LRH-1 expression in androgen-sensitive PCa cells is mediated through AR.

### Functional AR-binding sites are present and mapped to the regulatory regions of the *NR5A2* gene

**Figure 5:**
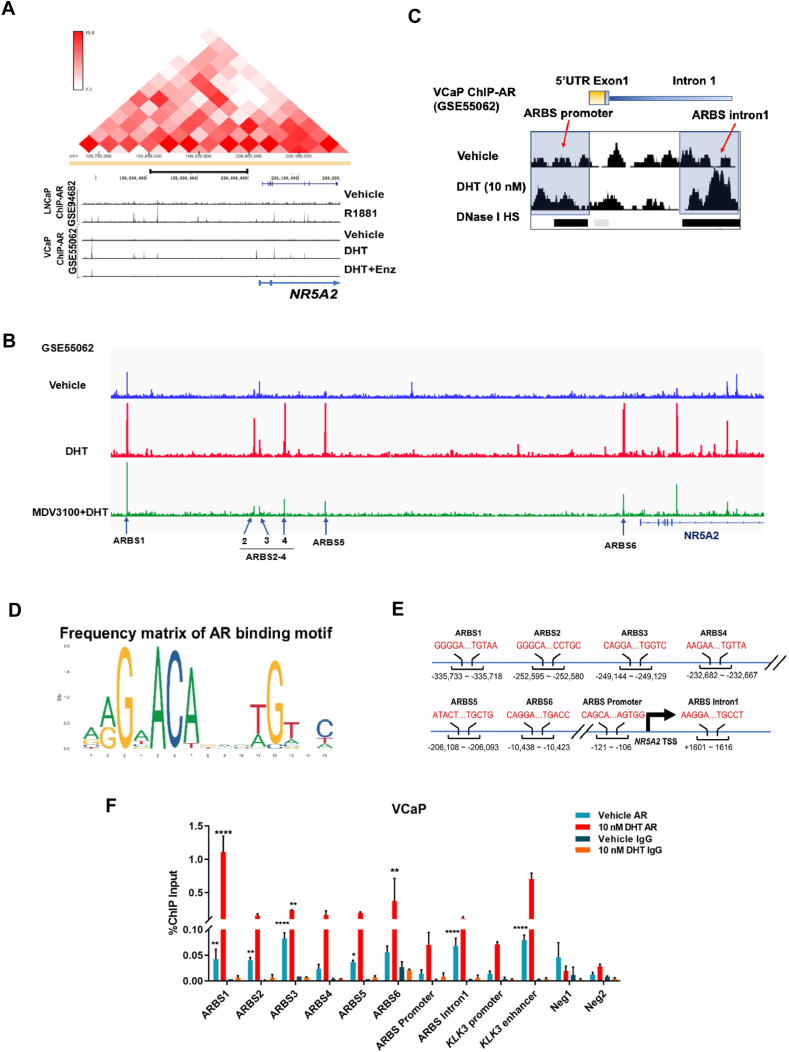
Functional AR-binding sites are present and mapped to the regulatory regions of the *NR5A2* gene. **(A)** The pyramid heatmap, generated from the Hi-C data of LNCaP cells and annotated using the Hi-C browser (ENCODES developed by the Dekker Laboratory), delineates the TAD of *NR5A2*. The AR ChIP-seq data derived from two GEO datasets (GSE94682 and GSE55062) were visualized using the UCSC Genome Browser (http://genome.ucsc.edu/cgi-in/hgGateway) and aligned with Hi-C data. Multiple ARBSs were identified within the *NR5A2* TAD, and their binding sites showed consistency in both LNCaP and VCaP cells. Their binding intensities could be stimulated by AR agonists (R1881 or DHT) and/or impaired by the AR antagonist, enzalutamide. **(B)** Mapping of six ARBSs (1–6) identified with strong AR binding signals within the enhancer region −400kb to the transcription start site of *NR5A2* using the AR ChIP-seq data from the R1881-treated (GSE84432) and DHT-treated (GSE55062) VCaP cells. Five strong and consistent ARBSs were commonly found in the *NR5A2* TAD in both AR ChIP-seq datasets, whereas ARBS4 was found to be DHT-specific. **(C)** Two ARBSs were identified in the promoter (ARBS-promoter) and intron 1 (ARBS-intron 1) of *NR5A2* using the AR ChIP-seq dataset of GSE55062 and analyzed via the UCSC Genome Browser. DNase I HS: DNase I hypersensitive sites. **(D)** The AR-binding motifs in the identified ARBSs and their frequency matrix were analyzed using the JASPAR database of transcription factor-binding motifs (http://jaspar.genereg.net). The height of the nucleotide characters represents their relative frequency in the corresponding positions of the AR-binding motifs. The most frequent motif, GGAACGGAACGTGTTCT, is shown to be a canonical AR-binding motif that contains both a direct repeat and an inverted repeat. **(E)** The AR-binding motifs of the regulatory elements of *NR5A2* are mapped to the identified ARBSs (1–6, -promoter and -intron1). All ARBSs show co-occupancy with DNase I-hypersensitive sites that represent the open chromatin sites for transcription factor-binding. The AR-binding motifs are underlined and shown in red. The DNase I-hypersensitive sites are identified after searching the *DNaseI* Hypersensitive Site Master List from the ENCODE/Analysis (Data version: ENCODE Jan 2011 Freeze). **(F)** ChIP-PCR assay of AR binding to the identified ABRSs performed in DHT-treated VCaP cells. VCaP cells were treated with or without DHT (10 nM) for 12 h before their nuclei were collected for ChIP assay with AR-specific antibody or IgG used as a control. The immunoprecipitated chromatin was assayed by PCR using primers for ARBSs located in distal and proximal regions of *NR5A2*. The primers for the enhancer and promoter of KLK3 (PSA) were used as positive control, while the primers for Neg1 and Neg2 for non-AR target sites were used as negative control for the ChIP assay. The results showed that the AR-binding signals on the identified *NR5A2*-associated ARBSs were further enhanced by DHT treatment. The relative abundance of AR-binding is presented as the mean ± SD. ∗∗*P* < 0.01; ∗∗∗∗*P* < 0.0001.

To determine whether LRH-1 could be a direct transcription target of AR in PCa cells, we next sought to identify and map the potential AR-binding sites (ARBSs) in the *NR5A2* gene (LRH-1). We analyzed the high-throughput ChIP-seq (Hi-C) dataset of AR binding activity in LNCaP cells treated with AR agonists (R1881) (GSE94682) and the ChIP-seq dataset of AR activity in VCaP cells treated with DHT or DHT plus enzalutamide (GSE55062). Analysis of both the Hi-C and ChIP-seq datasets revealed that a well-distinguished genomic region of the AR-regulatory TAD was identified and mapped to the *NR5A2* gene, and multiple potential chromatin ARBSs were identified within the *NR5A2* TAD (Fig. 5A). Detailed analysis further revealed that the signals of ARBSs located within the *NR5A2* TAD could be significantly enhanced by stimulation with AR agonists (DHT or R1881) in LNCaP cells or impaired by combined DHT plus enzalutamide treatment in VCaP cells. Based on these findings, the ARBSs identified in the highly active chromatin-looping region (−400 kb to +5 kb) within the *NR5A2* TAD were considered as the candidate AR regulatory elements (distal enhancers and promoters) of *NR5A2,* and their interactions were considered highly active based on the heatmap of Hi-C (Fig. 5A).

To further narrow down and validate the functionality of the identified ARBSs in the *NR5A2* TAD, we identified six ARBSs as potential genuine ARBSs that function in the *NR5A2* gene via comparison with the ChIP-seq dataset in VCaP cells treated with or without DHT (GSE55062) (Fig. 5B). Besides the six ARBSs (1–6) mapped in the distal enhancer regions, two proximal ARBSs (promoter and intron 1) were also identified in the promoter region (ARBS promoter) and intron 1 of the *NR5A2* gene (Fig. 5C). Among these, ARBS4 was regarded as the DHT-specific responsive site, as it could be induced by DHT-activated but not R1881-activated AR (likely due to different AR conformations induced by different AR agonists^45^^,^^46^) (Fig. 5A). We further analyzed the sequences of the identified ARBSs through matching with the position frequency matrix of AR-binding motifs using an open-access database of transcription factor-binding motifs JASPAR.^47^ Sequence analysis validated the presence of AR-binding motifs in all the six distal ARBSs and the two proximal ARBSs. Moreover, all the identified ARBSs were characterized to be located within or near the DNase I-hypersensitive sites (Fig. 5C), which are regarded as the accessible DNA or open chromatin regions for transcription factor binding. The AR-binding motif analysis validated the presence of androgen-response elements (AREs) in the ARBSs *in silico* (Fig. 5D and E). Finally, we performed a ChIP-PCR assay of AR on all the identified ARBSs. In accordance with the ChIP-seq data, the results showed that all the ARBSs displayed distinguishable AR-binding signals, with the signals further enhanced by DHT treatment (Fig. 5F). Taken together, multiple distal and proximal ARBSs containing AR-binding motifs were identified and mapped within the active chromatin/DNA-interaction domain of the *NR5A2* TAD, and their functionality was dependent on AR binding.

### AR binding to regulatory elements in the enhancer and promoter regions of *NR5A2* can induce an androgen-dependent chromatin looping pattern that impairs its transcription

**Figure 6:**
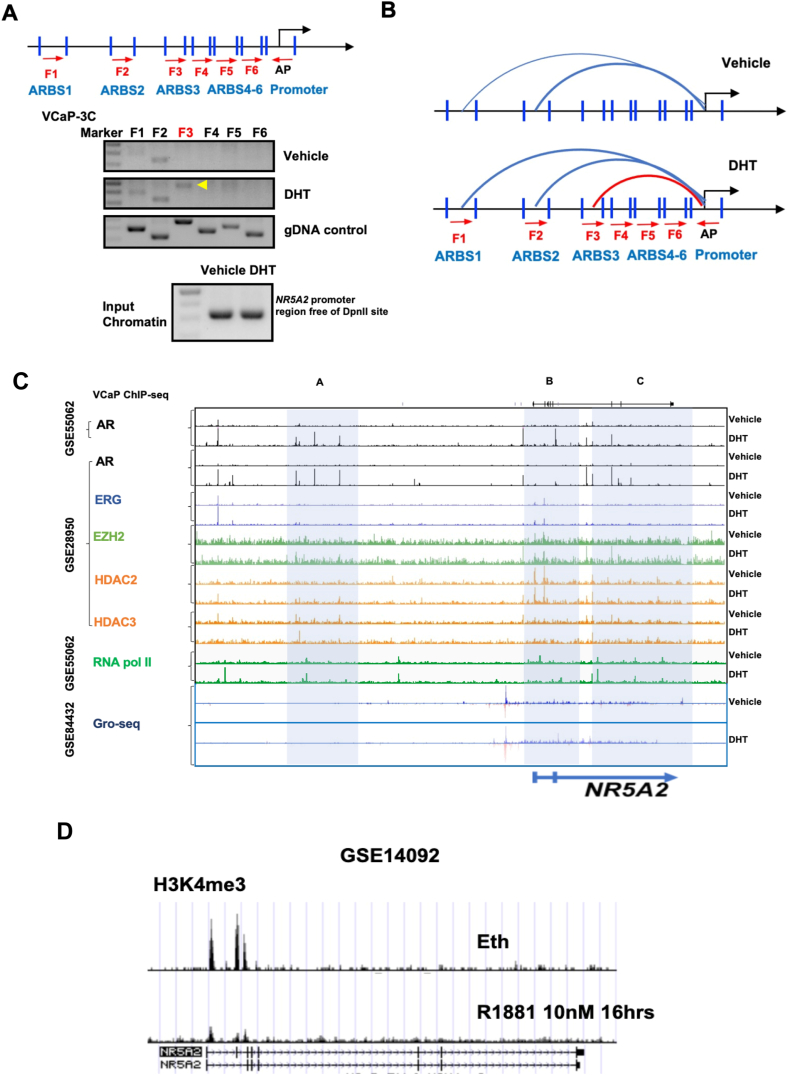
AR binding to enhancers and promoters of *NR5A2* can induce an androgen-dependent chromatin looping pattern. **(A)** Upper panel: schematic diagram shows the locations of primers designed around the regulatory elements of *NR5A2* and used for the 3C assay performed in VCaP cells. The blue peaks represent the *Dpn*II cut sites; red arrows represent the primer locations and directions; AP represents the anchor primer at the promoter and is used in pair with other ARBS enhancer primers F1–F6 for the 3C assay. Lower panel: 3C assay of *NR5A2* performed in AR-positive VCaP cells. The results showed that three PCR products were amplified in re-ligated chromatin using the F1–F3 enhancer primers and the AP anchor primer, with the F3-AP PCR product only amplified (marked by a yellow arrowhead) and the F1-AP PCR product further amplified by DHT treatment. DNA fragments encompassing the ARBSs and flanking two *Dpn*II cut sites were amplified, digested and re-ligated as random ligation products (gDNA control) and used as positive control of PCR. NR5A2 promoter DNA regions free of *Dpn*II cut sites were used as quality control for 3C-PCR. **(B)** Schematic diagrams show the interactions between the ARBSs in the distal enhancer and proximal promoter regions within the *NR5A2* TAD based on the 3C results. Under androgen-deprivation conditions, both ARBS1 and ARBS2 show interactions with the NR5A2 promoter, with ARBS2 as the predominant interaction site and ARBS1 as the weaker site. Upon DHT treatment/activation, the ARBS1-promoter interaction is increased and the ARBS3-promoter interaction is induced to form an androgen-dependent chromatin loop within the NR5A2 TAD. The semicircle lines represent the ARBS-promoter interactions, and the thickness of the line represents the relative intensity of the interaction. **(C)** Upper panel: analysis of the binding profiles of AR, ERG, EZH2, HDACs and the RNase pol II using ChIP-seq datasets derived from DHT-treated VCaP cells (10 nM, GSE55062; 100 nM, GSE55062). The results revealed that the DHT-induced binding peaks of AR, ERG, HDAC2/3 and EZH2 were co-occupied with the induced binding peaks of RNA pol II. RNA pol II showed more pausing peaks in the distal enhancer (highlighted zone A) and promoter regions (zone B) of NR5A2, whereas fewer binding peaks were observed in the gene body (zone C) upon DHT treatment compared with vehicle treatment. Lower panel: analysis of GRO-seq datasets derived from DHT-treated VCaP cells (100 nM, 4 h, GSE84432). The results showed that nascent RNA synthesis in the distal enhancer regions and proximal promoter region was dramatically suppressed by DHT treatment. **(D)** Analysis of the binding profile of histone H3K4me3 using a ChIP-seq dataset derived from R1881-treated VCaP cells (10 nM, 16 h, GSE14092). Analysis showed that the intensity of the H3K4me3 peak in exon 1, exon 2 and intron 2 of *NR5A2* showed significant decrease upon R1881 treatment.

The mapping of multiple distal ARBSs and the demonstration of a highly active DNA interaction domain formed between the distal regions (−400 kb) to the transcription start site of *NR5A2*, as revealed by the Hi-C data, suggest that the distal and proximal ARBSs could form regulatory chromatin loops via a functional transcription complex recruited by activated AR (Fig. 5A). To demonstrate the chromatin looping between the distal and proximal ARBSs of *NR5A2*, we then performed the 3C assay in VCaP cells. The results showed that three PCR products could be amplified in the chromatin re-ligated between the distal regions ARBS1-3 (primed by primers F1–F3) and the promoter (primed by primer AP). Among these PCR amplification signals, the signal of the F1-AP PCR product could be amplified and enhanced upon DHT treatment but attenuated by enzalutamide treatment, while the F2-AP PCR signal was maintained at a high level with no change upon DHT or enzalutamide treatment. Notably, the F3-AP PCR signal was only detected and amplified upon DHT treatment (Fig. 6A; Fig. S3A). These results suggest that the distal DNA regions spanned by ARBS1 and ARBS2 constantly interact with the promoter of *NR5A2* to form AR-dependent chromatin loops with such interaction loops further enhanced by DHT and that the chromatin loop formed by ARBS3 and promoter interaction can be induced specifically by DHT. Collectively, these results suggest a molecular mechanism of the AR-mediated repression on *NR5A2* via the formation of distinct patterns of chromatin looping between the distal enhancers and promoters in an androgen-dependent manner (Fig. 6B).

To further elucidate the molecular mechanism involved in the AR-mediated suppression on *NR5A2* transcription, we analyzed the ChIP-seq data of AR, ERG, EZH2, HDACs and RNA polymerase II (RNase pol II) in DHT-treated VCaP cells (GSE55062 and GSE28950), and observed that AR, ERG (acting as a repressor of AR signaling) and other commonly over-expressed co-repressors (EZH2, HDAC2 and HDAC3) were co-localized within the distal enhancers and/or proximal promoters of *NR5A2* (Fig. 6C). Moreover, in consistency with the suppressive effect of androgen on *NR5A2* mRNA expression, the ChIP-seq data (GSE55062) revealed that there were more paused peaks in the enhancer and promoter regions of *NR5A2* and fewer RNA pol II peaks in the gene body upon DHT treatment, and the paused peaks also showed frequent co-occupancy with AR binding sites (Fig. 6C), further supporting the suppression effect of androgen-activated AR on the RNA pol II-mediated mRNA synthesis of *NR5A2*. To demonstrate the status of nascent RNA synthesis in *NR5A2*, we next analyzed the GRO-seq data of DHT-treated VCaP cells (GSE84432). The results revealed that the nascent RNA synthesis on the distal enhancer and proximal promoter regions of *NR5A2* decreased upon DHT treatment (Fig. 6C). Furthermore, analysis of ChIP-seq profiles of histone H3K4me3 in VCaP cells revealed that H3K4me3 enrichment in exons 1 and 2, and intron 2 of *NR5A2* dramatically decreased upon R1881 treatment (Fig. 6D, GSE14092), suggesting that histone methylation was involved in the androgen- or AR-mediated suppression of *NR5A2*.

**Figure 7:**
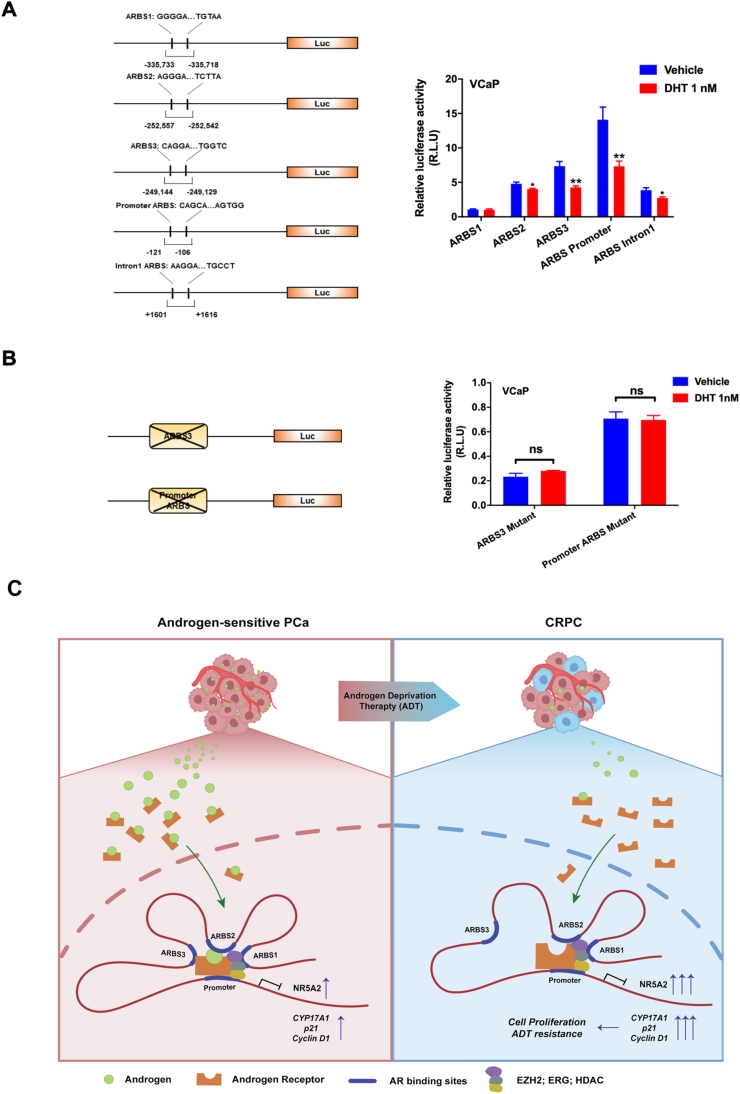
AR signaling mediates transrepression of the *NR5A2* gene. **(A, B)** Luciferase reporter assay of regulatory regions of the *NR5A2* gene performed in AR-positive VCaP cells. (A) Left panel: Schematic diagram shows the five reporter constructs driven by different ARBS-containing DNA fragments derived from the enhancers, promoter and intron 1 of *NR5A2*. Right panel: The results showed that the luciferase activities of the ARBS3 and ARBS-promoter reporters were significantly repressed, while those of the ARBS2 and ARBS-intron1 reporters were moderately/slightly repressed by DHT treatment. (B) Left panel: Schematic diagram shows two mutant reporters with truncations of ARBS-binding motifs. Right panel: The results showed that deletions of ARBSs in ARBS3 and ARBS-promoter reporters abolished their DHT-induced luciferase activities. **(C)** Schematic diagram shows the proposed model of the androgen-induced dynamic conformational change in chromatin looping within the *NR5A2* TAD. Upon DHT treatment, a new chromatin loop is formed by the interaction between ARBS3 and the promoter of *NR5A2*, with the recruitment of other corepressors or epigenetic modifiers, including ERG, EZH2 and HDACs, which may favor the suppression on the *NR5A2* transcription. ∗*P* < 0.05; ∗∗*P* < 0.01; ns: not significant.

Finally, to further demonstrate the direct transrepression of *NR5A2* by AR in PCa cells, we performed the reporter gene assays in AR-positive VCaP cells using multiple ARBS-containing luciferase reporter constructs. The results showed that the luciferase activities of reporters driven by fragments containing ARBS2, ARBS3, the promoter and intron 1 could be decreased by DHT treatment, while the luciferase activities of reporters driven by ARBS1, ARBS2, ARBS3 and the promoter region could be increased by CS-FBS or combined CS-FBS and enzalutamide treatment (Fig. 7A; Fig. S3B). Among these reporters, the ARBS3- and promoter-containing reporters exhibited higher suppression than the other reporters by DHT treatment, suggesting that AR could mainly suppress *NR5A2* transcription via its direct binding to ARBS3 and promoter-containing regions. Deletion of the ARBS3 and ARBS-promoter elements in these reporters abolished the DHT-induced suppression effect, further confirming that these binding motifs were essential for the AR-mediated transrepression of *NR5A2* (Fig. 7B). Collectively, these results demonstrate a molecular mechanism underlying the androgen-induced suppression of *NR5A2* transcription that is mediated through the formation of distinct chromatin loops in the TAD of *NR5A2* formed by AR binding to the enhancer and promoter regions, resulting in a reduction of RNA pol II-initiated mRNA synthesis of *NR5A2* or LRH-1 (Fig. 7C).

## Discussion

LRH-1 (*NR5A2*), serving as an oncogenic factor, has been implicated in multiple cancers via distinct regulatory pathways. Several synthetic LRH-1 modulators, such as ML-180 (LRH-1 specific inverse agonist), have been exploited during the past decade, suggesting the pharmacological potential of targeting LRH-1 in the management of various cancers.^48^ We previously showed that LRH-1, exhibiting an increased expression in clinical CRPC tissues and CRPC xenograft models, plays a critical role in the progression of CRPC via its promotion of intratumoral androgen biosynthesis.^8^ However, the mechanisms that contribute to its increased expression in CRPC are still not well understood. In the present study, we further confirmed the up-regulation pattern of LRH-1 in two additional CRPC xenograft models, as well as our PDX-derived CRPC and androgen-insensitive LNCaP subline models. We also observed that LRH-1 expression could be suppressed by androgen but conversely up-regulated by antiandrogen in PCa cells, suggesting a negative role of AR signaling in the regulation of LRH-1 expression in PCa and thus helping to explain our previous observation that LRH-1 was sharply up-regulated in hosts upon castration (with acute down-regulation of AR signaling).^8^ We also detected LRH-1 protein expression in multiple AR-positive (VCaP, LNCaP and 22Rv1) and AR-negative (PC3 and DU145) PCa cell lines. Among the AR-positive cell lines, 22Rv1 exhibited weaker levels than VCaP (wild-type AR) and LNCaP (AR with a point mutation in the ligand-binding domain), probably related to its dual androgen-responsive and androgen-insensitive features and the expression of multiple AR splice variants, particularly the AR-V7 with LDB truncation^49^; however, LRH-1 expression in AR-negative cells could be regulated by non-AR regulators yet to be identified.

Recent Hi-C mapping has revealed substructure within chromatin compartments termed as topologically associating domains (TADs), which facilitate interactions between *cis*-regulatory elements and their target promoters and thus have been perceived as structural scaffolds for regulatory landscapes.^23^^,^^50^ Emerging evidence suggest that in addition to functioning as a transactivator, AR also acts as a repressor of its own gene and other genes in prostate cancer via a transcription mechanism involving chromatin looping between the enhancer and promoter regions.^17^^,^^51^ In this report, through detailed genomics and molecular analyses, we identified multiple distal and proximal ARBSs mapped within the active chromatin/DNA-interaction domain of the *NR5A2* TAD, in which AR could transrepress the *NR5A2* gene via the formation of distinct patterns of chromatin looping between its distal enhancers and promoter in response to androgen stimulation or an antiandrogen. Notably, transcriptional corepressors of AR are often aberrantly over-expressed in PCa. TMPRSS2: ERG (T: E) is the most common form of ETS gene fusion identified in CRPC.^52^ Previous studies have indicated that ERG can bind to the ARBSs of the androgen-regulated genes and mediate the inhibition of androgen-dependent transcription.^30^ Besides, ligand-activated AR has been shown to mediate the assembly of the repressive complexes at the ARE-containing element of the AR-repressed gene by recruiting HDACs, LSD1, or EZH2, which results in histone modifications at the local chromatin, thus impeding the accessibility of regulatory elements.^15^^,^^30^ In line with these findings, by dissecting several ChIP-seq data, we found that AR, ERG, EZH2 and HDACs can be co-localized to the distal enhancers and/or proximal promoters of *NR5A2* upon AR activation. On the contrary, the level of active marks such as RNase pol II and H3K4me3 are significantly reduced in response to androgen stimulation. These results suggest that transcriptional corepressors or epigenetic regulation may be involved in the suppressive effect of androgen-activated AR on LRH-1. It will be of great interest to determine the specific corepressors contributing to AR-mediated suppression of *NR5A2* (LRH-1) in future studies.

It has been well characterized that AR has both growth-promoting and growth-inhibiting effects on PCa cells.^15^ In this study, we also showed that high doses of androgen could suppress the cell proliferation and the expression of LRH-1 in androgen-responsive PCa cells via the direct transcription repression by AR. Indeed, a wide range of previous studies have shown that high or physiological levels of androgen can inhibit the *in vitro* growth of androgen-responsive PCa cells and their *in vivo* xenograft growth; conversely, low or castrate levels of androgen can promote both *in vitro* and *in vivo* tumor growths in castrated hosts.^53^^,^^54^ However, the extent to which androgen levels in CRPC stimulate AR functioning as either a transcription activator or repressor via its differential recruitment of coactivators or epigenetic (histone) modifiers is still unclear and needs further investigation. Interestingly, a previous study showed that androgen levels in CRPC cells are sufficient to activate AR on enhancer elements of genes governing several critical metabolic functions (e.g., lipid synthesis), which are sensitive to lower levels of androgens but are inadequate to recruit AR to suppressor elements in genes that negatively regulate cellular proliferation; AR can function to repress its gene expression and the expressions of two steroidogenic enzymes (AKR1C3 and HSD17B6), and a negative feedback regulatory loop is formed to suppress AR signaling at high androgen levels, but this negative loop can allow increased AR expression and androgen biosynthesis in CRPC.^17^ Based on our current findings, we hypothesize that decreased androgen levels in CRPC may relieve the AR-repressed genes involved in intratumoral androgen biosynthesis, including LRH-1, and thereby maintain a status of androgen homeostasis and reactivation of AR signaling, contributing to CRPC growth and therapy resistance.

In summary, our present study elucidates an AR-dependent mechanism that contributes to increased *NR5A2* gene expression and compensatory androgen homeostasis in CRPC and identified that a promoter-enhancer DNA looping formed within the TAD of *NR5A2* is mediated by direct binding of AR to the regulatory elements for AR-mediated transcriptional repression of *NR5A2*. Notably, several small-molecule inhibitors or inverse agonists targeting LRH-1 have been developed.^55^ Besides, recent technological advancements in the development of proteolysis-targeting chimera (PROTAC) and peptidomimetics also show a promise in drugging the ‘undruggable’ transcription factors in cancers.^56^ Therefore, our results also suggests that simultaneous or sequential combination of targeting LRH-1 and ADT could be a potential therapeutic strategy for the management of CRPC.

## CRediT authorship contribution statement

**Wenxing You:** Writing – original draft, Visualization, Software, Methodology, Formal analysis, Conceptualization. **Tiantian Gao:** Writing – review & editing, Visualization, Validation, Software, Methodology, Formal analysis. **Haolong Li:** Resources, Methodology, Formal analysis. **Daniel Hau Tak Lam:** Formal analysis, Methodology. **Wenjuan Xie:** Resources, Methodology, Formal analysis. **Lijia Xiao:** Resources, Methodology, Funding acquisition, Conceptualization. **Weijie Gao:** Methodology, Conceptualization. **Dinglan Wu:** Methodology, Funding acquisition, Conceptualization. **Junjian Wang:** Methodology, Conceptualization. **Yuliang Wang:** Writing – review & editing, Writing – original draft, Resources, Methodology, Funding acquisition, Conceptualization. **Franky Leung Chan:** Writing – review & editing, Supervision, Funding acquisition.

## Ethics declaration

All animal procedures were performed according to the institutional laboratory animal guidelines and with approval (22-300-GRF) from the CUHK Animal Experimentation Ethics Committee.

## Funding

This work was supported by the 10.13039/501100001809National Natural Science Foundation of China (No. 81802575 and 82072830 to YW; 81974457 to LX; 81872283 to DW), the Science and Technology Project of Shenzhen (No. JCYJ20210324130607021) to YW, the 10.13039/501100003453Natural Science Foundation of Guangdong Province, China (No. 2019A1515012079) to LX; and grants from the Health and Medical Research Fund (No. 02130066), Food and Health Bureau of Hong Kong; General Research Fund (No. 14107623), Research Grants Council of Hong Kong to FLC.

## Conflict of interests

The authors have no conflict of interests in this study.
