# Supplementary material for: Androgen receptor acts as the transcriptional repressor of the nuclear receptor LRH-1 via the androgen-driven chromatin looping conformation in prostate cancer
Source: Genes Dis. 2025 Oct 24;13(4):101903. doi: 10.1016/j.gendis.2025.101903 (PMC13090603; doi:10.1016/j.gendis.2025.101903)
Supplement: Multimedia component 1 [file mmc1.pdf]

Androgen receptor acts as the transcriptional repressor of nuclear receptor LRH-1 via androgen-driven chromatin looping conformation in prostate cancer

Wenxing You, Tiantian Gao, Haolong Li, Daniel Hau Tak Lam, Wenjuan Xie, Lijia Xiao, Weijie Gao, Dinglan Wu, Junjian Wang, Yuliang Wang and Franky Leung Chan

Supplementary information of Materials and Methods

Supplementary Table S1

Culture media for cell lines used in this study

Cell lines	Culture media for cell growth
LNCaP	RPMI 1640 with 4.5g/L glucose, 1 mM sodium pyruvate, 10 mM HEPES and 10% FBS
LNCaP-AI	RPMI 1640 with 10% charcoal-stripped serum
VCaP	DMEM with 10% FBS
DU145	MEM with 1 mM sodium pyruvate and 10% FBS

Supplementary Table S2

Core sequences used for shRNA plasmid construction:

Target	Nucleotide sequences
AR	AAGCTCAAGGATGGAAGTGCAGTTAGGGCTG
Scramble	CCGTACCTACACGCAGCGCTGACAACAGTTT

Supplementary Table S3

Primer pairs and restriction enzymes used for luciferase reporter construction:

Targets	Nucleotide sequences of primers	
ARBS1	Forward	agagaGCTAGCAAGCCTCCCTCAGAGGTCAT
	Reverse	agagaCTCGAGGGGGCAGACCTCATGGTTT
ARBS2	Forward	agagaGCTAGCTGACAGTGGTTGCCTAGAAGAG
	Reverse	agagaCTCGAGCTGTTTGACTTCTCCCCGTTT
ARBS3	Forward	agagaGCTAGCCTGCAACTCTTCCTGCCCTC
	Reverse	agagaCTCGAGAGTCTGCGCCTTATCTCTCA

ARBS-promoter	Forward	agagaGGTACCGAGCTTGGAAATGCTACCTGTAG
	Reverse	agagaGCTAGCGTGACCTGCTGTAAACCATG
ARBS-intron1	Forward	agagaGCTAGCGTCTGTTGGAAGTCAGGCCC
	Reverse	agagaCTCGAGTCGTGGAGGCTACGAGGTAA
ΔARBS3-deletion	Forward 1	agagaGCTAGCCTGCAACTCTTCCTGCCCTC
	Reverse 1	CTCACTTCATGGGCGGGTTAGCTTTTG
	Forward 2	ACCCGCCCATGAAGTGAGGTCTGAGGTCA
	Reverse 2	agagaCTCGAGAGTCTGCGCCTTATCTCTCA
ΔARBS-promoter deletion	Forward 1	CTAGCAAAATAGGCTGTCCCCAGTG
	Reverse 1	GTGGAGAGGTGCTTAAAACTGAGGGGTCAGAAGATCAG
	Forward 2	CCCTCAGTTTTAAGCACCTCTCCACTTCTGAGTTAATCATTTC
	Reverse 2	CTTTATGTTTTTGGCGTCTTCCATGGTG

Supplementary Table S4

Primer pairs used for SYBR-based qPCR assay:

Gene targets	Nucleotide sequences		GenBank accession No.
ACTB (β-actin)	Forward	ATGGATGATGATATCGCCGCG	NM_001101
	Reverse	CTCCATGTCGTCCCAGTTGGT	
NR5A2 (LRH-1)	Forward	TCGACCACATTTACCGACAAG	NM_205860;<br>NM_003822;
	Reverse	CCACTAACTCCTGTGCATGACT	NM_001276464
AR	Forward	CGGAAGCTGAAGAAACTTGG	NM_000044
	Reverse	ATGGCTTCCAGGACATTCAG	
KLK3 (PSA)	Forward	TTGTCTTCCTCACCCTGTCC	NM_001648
	Reverse	TCACGCTTTTGTTCCTGATG	

Supplementary Table S5

Primer pairs used for ChIP-PCR assay:

Gene targets	Nucleotide sequences	
ARBS1	Forward	AACTACAGCTCCAACACCACTTT
	Reverse	TTTTGATGCAAGGGCTTGACTACAG

ARBS2	Forward	CCTCCACCTATGAGGTAAGCAAGT
	Reverse	GTGCTTACCAGACTTCTCCTGG
ARBS3	Forward	ACACAAAAGCTAACCCGCCCA
	Reverse	ATGCCCCACAAATGACCTCAGA
ARBS4	Forward	TAACTGGCACAGTTGAGTGCCAC
	Reverse	TGGGATTGGTATTTGTAATGATGATGCAAAAG
ARBS5	Forward	GCCCAGGATCTATGTTGAGACA
	Reverse	TCTTGGCAATACGTGGGAAC
ARBS6	Forward	AGGAGAAAAGACTAGGAGCCAAACTGGC
	Reverse	ATTGAGCTGGGACCAGATCTGGCTTC
ARBS-promoter	Forward	AGTTAGCTGGTTTGCAGTTTT
	Reverse	AGTGGAGAGGTGCTATGCAG
ARBS-intron1	Forward	GCAGGGTGTAACACACAATCAA
	Reverse	GCCCGACGAAAAAGATGCGA
PSA-enhancer	Forward	GCCTGGATCTGAGAGAGATATCATC
	Reverse	ACACCTTTTTTTTTCTGGATTGTTG
PSA-promoter	Forward	CCTAGATGAAGTCTCCATGAGCTACA
	Reverse	GGGAGGGAGAGCTAGCACTTG
Negative 1	Forward	TCATCATGAATCGCACTGTTAGC
	Reverse	GCCCAAGTGCCTTGGTATACC
Negative 2	Forward	CAGAGGGCTTCTGGTGAAAC
	Reverse	TTGACAATGTCTTGCCTTGG

Supplementary Table S6

Primer pairs used for 3C-PCR assay:

primers for ChIP-qPCR	Nucleotide sequences	PCR size	Distance to NOV TSS	Distance to Nearest DpnII
AP	AGGCCACGAAATTTGACAAGC	NA	+224	173
F1	GCCAACACACACACTATTTCCAC	290	-335,609	117
F2	ACAAAGATGGTTCTAGCCTCTAGC	219	-252,286	46
F3	CTGCAGCTTTAACTGCTACAAGG	375	-249,036	202

F4	TTCGCCATTGCTTTTGCATCATC	278	-232,495	105
F5	GATTGGCCTACCTGTCCTCT	321	-205,150	148
F6	GCCAGTTTGGCTCCTAGTCTTTTC	251	-10,486	78
gDNA control F	AATTCTTATCTCTTTTGCTGTCACT	295	53	
gDNA control R	GCTTGTCAAATTTCGTGGCCT	295	242	

Supplementary Methods details

Generation of stable AR-knockdown VCaP-shAR transductants

Lentiviral plasmids, pLKO.1-puro-shAR expressing AR-targeting shRNA and pLKO.1-puro-shScramble expressing scramble sequence as negative control, were generated. shRNA lentiviruses were packaged in 293FT packaging cells and used to infect VCaP cells for either stable or transient gene knockdown studies. Cells were infected with lentiviruses together with hexadimethrine bromide for 24 hr, followed by puromycin selection for 8 days.
