# Supplementary material for: Androgen receptor acts as the transcriptional repressor of the nuclear receptor LRH-1 via the androgen-driven chromatin looping conformation in prostate cancer
Source: Genes Dis. 2025 Oct 24;13(4):101903. doi: 10.1016/j.gendis.2025.101903 (PMC13090603; doi:10.1016/j.gendis.2025.101903)
Supplement: Multimedia component 2 [file mmc2.pdf]

Androgen receptor acts as the transcriptional repressor of nuclear receptor LRH-1 via androgen-driven chromatin looping conformation in prostate cancer

Wenxing You, Tiantian Gao, Haolong Li, Daniel Hau Tak Lam, Wenjuan Xie, Lijia Xiao, Weijie Gao, Dinglan Wu, Junjian Wang, Yuliang Wang and Franky Leung Chan

Supplementary Figures 1-3

Supplementary Figure 1. RT-qPCR analysis of LRH-1 expression in DU145 cells upon treatment with DHT.

Supplementary Figure 2. (A) RT-qPCR analysis of LRH-1 expression in VCaP cells upon combined treatment with DHT and enzalutamide. **(B)** CCK-8 assay in DU 145 cells treated with DHT

Supplementary Figure 3. (A) 3C assay of NR5A2 performed in AR-positive VCaP cells treated with Enzalutamide. **(B)** Luciferase report assay of regulatory regions of NR5A2 gene performed in AR-positive VCaP cells treated with either enzalutamide (ENZ) or charcoal stripped serum (CS-FBS).

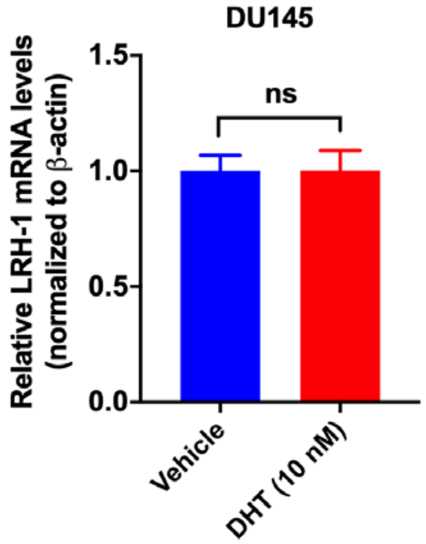

Supplementary Figure 1. RT-qPCR analysis of LRH-1 expression in DU145 cells upon treatment with DHT. DU145 cells were pre-cultured with CS-FBS medium for 72 hours before treatment with 10nM DHT for 24 hours. Results showed that no significant change of LRH-1 mRNA level was detected in DU145 cells upon DHT treatment. ns: not significant.

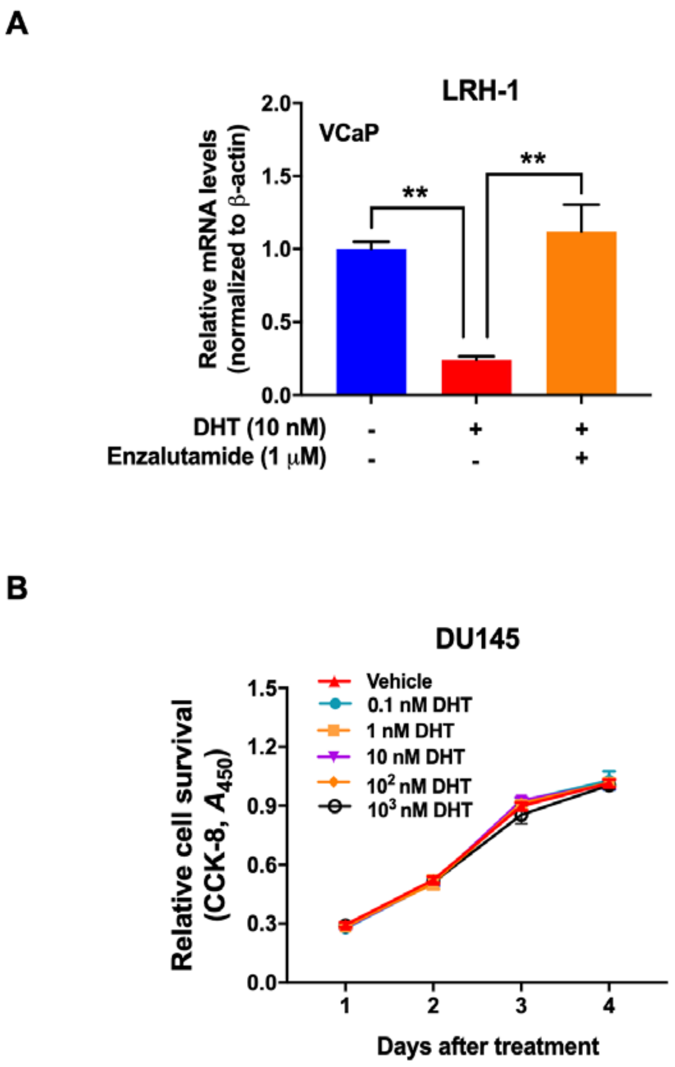

Supplementary Figure 2. (A) RT-qPCR analysis of LRH-1 expression in VCaP cells upon combined treatment with DHT and enzalutamide. VCaP cells were pre-cultured with CS-FBS medium for 72 hours before treatment with either DHT alone or combined DHT and enzalutamide for 24 hours. Results showed that the DHT-induced suppression on LRH-1 expression could be completely abolished by combined DHT and enzalutamide in VCaP cells. **(B)** CCK-8 assay. AR-negative DU145 cells grew at comparable rates in culture with DHT of doses 0.1-10^3 nM. **P < 0.01.

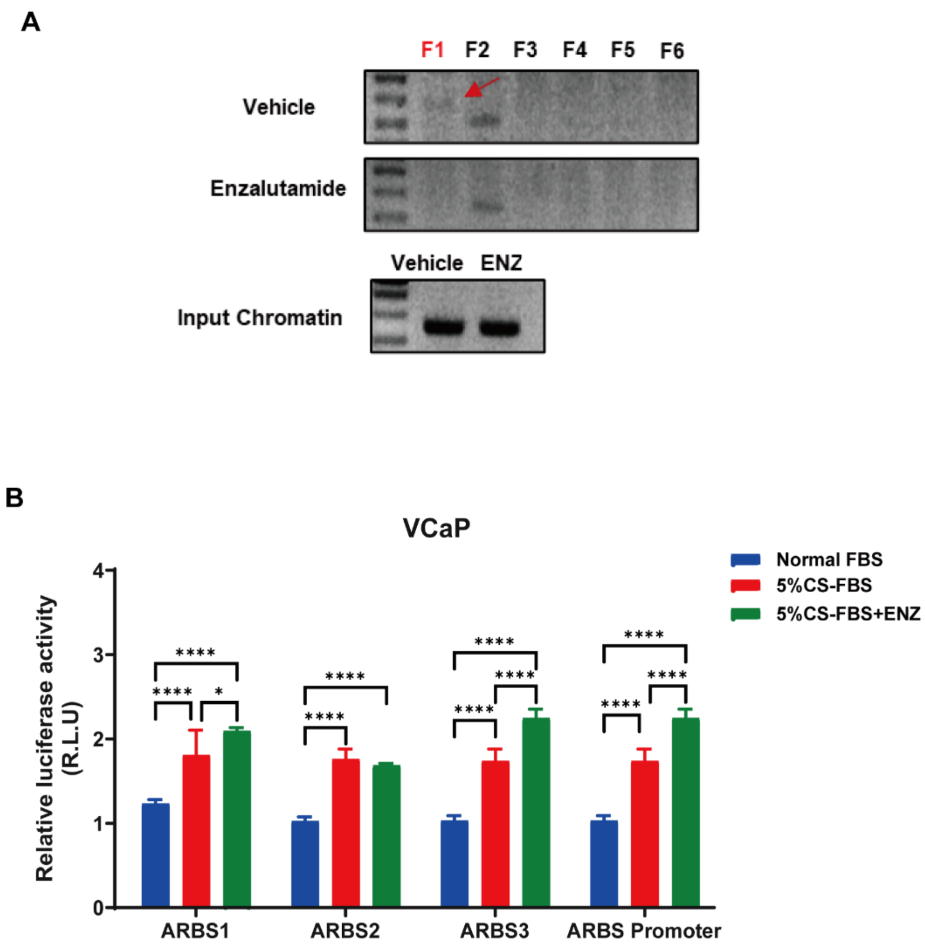

Supplementary Figure 3. (A) 3C assay of NR5A2 performed in AR-positive VCaP cells treated with enzalutamide. Results showed that the PCR product was decreased in re-ligated chromatin using the F1 enhancer primers and AP anchor primer (marked by red arrowhead) after enzalutamide treatment, suggesting ARBS1/ARBS2-promoter chromatin loops could be weakened by enzalutamide treatment. NR5A2 promoter DNA regions free of *Dpn*II cut sites were used as quality control for 3C-PCR. **(B)** Luciferase report assay of regulatory regions of NR5A2 gene performed in AR-positive VCaP cells treated with either enzalutamide (ENZ) or charcoal stripped serum (CS-FBS). Results showed that activities of reporters driven by

fragments containing ARBS1, ARBS2, ARBS3 and promoter could be increased by enzalutamide or CS-FBS. **$P < 0.01$.
